# Are people in the bush really physically active? A systematic review and meta-analysis of physical activity and sedentary behaviour in rural Australians populations

**DOI:** 10.7189/jogh.10.010410

**Published:** 2020-06

**Authors:** Carlos Ivan Mesa Castrillon, Paula R Beckenkamp, Manuela L Ferreira, Jose A Michell, Vania Alice de Aguiar Mendes, Georgina M Luscombe, Emmanuel Stamatakis, Paulo Henrique Ferreira

**Affiliations:** 1 The University of Sydney, Discipline of Physiotherapy, Faculty of Medicine and Health, Sydney, Australia; 2 Institute of Bone and Joint Research, The Kolling Institute, Northern Clinical School, Faculty of Medicine and Health, The University of Sydney, Sydney, Australia; 3 University of Sao Paulo, Department of Neuroscience and Behavioral Sciences, Sao Paulo, Brazil; 4 The University of Sydney, School of Rural Health, Faculty of Medicine and Health, Orange, Australia; 5 The University of Sydney, Charles Perkins Centre, Faculty of Medicine and Health, School of Health Sciences, Sydney, Australia

## Abstract

**Background:**

Physical inactivity is a major risk factor for non-communicable disease and premature mortality. People who live in rural settings are usually regarded as more physically active than those living in urban areas, however, direct comparisons between these populations are scarce. We aimed to summarise the prevalence of physical inactivity and sedentary behaviour in rural settings in Australia, compared to urban counterparts.

**Methods:**

We searched six databases (AMED, Embase, Medline; CINAHL, SPORTDiscus; and RURAL) and identified 28 observational studies that investigated the levels of physical inactivity and/or sedentary behaviour in adults aged 18 years and over in rural Australia. Random effects meta-analysis was used to generate pooled prevalence estimates.

**Results:**

Physical inactivity was four percentage points (95% confidence interval (CI) = 0.4 to 8) higher in rural populations compared to urban populations. There was a one percentage point (95% CI = -3 to 5) prevalence difference of physical activity in the rural populations. Rural populations reported on average 7.8 hours of sedentary time per day (95% CI = 5 to 10) and the prevalence of high levels of sedentary behaviour (≥to 8 hours per day) was 7% (95% CI = -8 to -7) greater in urban areas compared to rural areas.

**Conclusions:**

People living in rural areas are just as physically inactive as people who live in urban areas. Our findings challenge the popular views that rural lifestyles result in people engaging more frequently in physical activity. Public health campaigns promoting physical activity in rural settings are just as necessary as in urban settings.

Physical activity is an important strategy to reduce the burden of non-communicable diseases. According to the World Health Organization (WHO), adults should accumulate at least 150 minutes of moderate-intensity aerobic physical activity, 75 minutes of vigorous-intensity aerobic activity, or an equivalent combination of both during the week to experience the health benefits of physical activity [1]. It is important to clarify that insufficient physical activity refers to people performing some physical activity but not enough to comply with the WHO recommendations, whilst, physical inactivity refers to the lack of physical activity and also includes people who are insufficiently active. Not meeting physical activity recommendations or having increased time spent in sedentary behaviour (eg, a low energy expenditure rate <1.5 Metabolic Equivalent of Task [MET] in a sitting or reclining posture during waking times) [2] are linked with higher mortality rates and a wide array of diseases including type II diabetes, cardiovascular diseases, and musculoskeletal pain [3].

It is estimated that in 2014, around 3.4 billion people (46% of the global population) resided in rural areas worldwide ^[^4^]^, and in 2017 approximately 7 million people (29% of the Australian population) resided in rural or remote areas of Australia [5]. Importantly, even though there is a large body of literature supporting the benefits of engaging in regular physical activity, the focus of studies addressing physical activity has been primarily in populations living in urban environments [6]. Meanwhile, people who live in rural areas are more prone to face significant obstacles to be physically active, including limited built environments or places available for exercise and regular active commuting [7]. These barriers could potentially impact negatively on the adoption of an active lifestyle, and the quality of life of those living in rural communities.

Therefore, a comparison of the prevalence of physical inactivity and sedentary behaviour in people living in rural and remote areas could highlight needs for strategies in underserved populations. However, no systematic review comparing physical inactivity and sedentary behaviour prevalence in people residing in rural areas have been conducted. The main aim of this study was to systematically review studies assessing the prevalence of physical inactivity and sedentary behaviour in rural Australia settings. A secondary aim was to make a comparison in physical inactivity levels and sedentary behaviour between rural and urban populations.

## METHODS

The review protocol was prospectively registered in PROSPERO (CRD42017078170), and the Preferred Reporting Items for Systematic Reviews and Meta-Analyses (PRISMA) statement guided reporting.

### Search strategy

Electronic searches were conducted on six databases (three via Ovid: AMED, Embase and Medline; two via EBSCO-host: CINAHL and SPORTDiscus; and one via Informit Online (1996-2006) RURAL: Rural and Remote Health Database) from inception to 25th March 2019. In addition, citation tracking of the included studies and relevant systematic reviews was conducted. No date or language restrictions were applied.

The search strategy combined Medical Subject Heading (MeSH) terms and keywords related to physical activity (eg, exercise, walk, swim, etc), sedentary behaviour (eg, sitting, inactive, etc), and rural areas and/or rural populations, based on previously published Cochrane systematic reviews including “physical activity”, “sedentary behaviour”, and “rural setting”. The complete search string can be found in Table S1 in the **Online Supplementary Document**.

### Study selection

Observational studies (ie, prospective or retrospective cohort, case-control, cross-sectional) that investigated the levels of physical activity and/or sedentary behaviour in adults aged 18 years and over, residing in rural or remote Australian regions were included (ie, studies that identified their populations as being adults from rural or remote regions). We accepted the study definition for adults and rural or remote regions provided by the individual study authors. Randomised controlled trials and literature reviews were excluded. We excluded studies that recruited clinical populations (eg, cancer, diabetes, pregnancy, etc.) and only samples of physically active or inactive populations.

Both subjective (eg, questionnaires) and objective (eg, accelerometers) measures of physical activity participation and sedentary behaviour were included. We excluded studies that did not provide data on the prevalence of physical activity in urban and rural populations separately (ie, we excluded studies when a single prevalence value of physical activity was averaged and presented for both populations).

All retrieved records were imported into Endnote X7 (Thomson Reuters). At the first stage of title screening, one reviewer (CMC) performed title screening and a second reviewer (JAM) screened a random sample of 200 titles. Agreement was found in 99% of records included and disagreements were resolved by consensus. In the second stage, two reviewers (CMC and JAM) screened the studies through the abstract and an independent reviewer (PRB) screened again a random sample of 200 studies from all records. In the final stage, one reviewer (CMC) performed full-text screening and an independent reviewer (VAA) screened a random sample of 200 studies from all records. Disagreements were resolved by consensus.

### Data extraction and risk of bias assessment

Two independent reviewers (CMC and VAA) extracted data using a standardized pre-piloted spreadsheet. Data extracted included: sample source and size, study design, participants’ age and sex, as well as anthropometric characteristics, authors’ definition of rural or remote areas, tool(s) employed to assess physical activity levels and/or sedentary behaviour, and data on physical activity and/or sedentary behaviour. When more than one study time point was reported, we used the baseline data in the analyses. We contacted 19 authors of potential studies to request the necessary data to be included in our systematic review (eg, where the study did not report the raw value of physical activity prevalence in a rural population), with three (16%) authors providing the necessary data for meta-analysis purposes.

In order to compare results between studies and to maximize the amount of data available for pooling, we adjusted the extracted data according to similar categories. The outcomes from the included studies were categorized as “physically inactive” if the outcome was reported as being 0 to 149 minutes per week, and ‘physically active’ if the outcome was reported as being ≥150 minutes per week, according to current guidelines [1]. For studies that did not specify what guideline was used to categorize physical activity outcomes, we used the WHO definitions to classify it according to the outcome reported (eg, self-reported results coded as active and very active, were considered as engaging in ≥150 minutes per week of moderate physical activity). Since some studies used different approaches to categorize or assess physical activity and physical inactivity, in this systematic review these variables are not complementary as a total percentage value consequently, it was necessary to report on both separately. We accepted the study authors’ definitions of sedentary behaviour. In the case of several categories of sedentary behaviour reported in a single study, we selected the value of ≥ to 8 hours per day of sedentary activities to categorise sedentary behaviour [8].

We accepted the studies’ definitions of rural or remote regions, and urban regions. Where studies reported physical activity data on more than one category of rurality (eg, ‘regional centre’, ‘large rural’ and ‘small rural’), the categories were collapsed into a new category coded as ‘rural’ and physical activity measures were calculated accordingly. We performed a sensitivity analysis to investigate the effect of different levels of remoteness (eg, less remote, more remote) on the prevalence of physical inactivity and physical activity. For this purpose, where studies reported data on more than one category of rurality (eg, “regional centre”, “large rural,” and “small rural”), the furthest locations to urban centres or smallest locations in terms of population size, occupied an independent category and were coded as “more remote” for the purposes of the meta-analysis. Meanwhile, the greatest or closest locations to urban centres (eg, “regional centre” and “large rural”) were collapsed into a new one category and were coded as “less remote”.

The risk of bias of included studies was assessed using a modified version of the Newcastle-Ottawa Scale (NOS) for Prospective Cohort Studies and was performed by two independent reviewers (CMC) and (VAA). The appropriateness of representativeness of the sample, selection of non-exposed cohort, adjustment for risk factors (age, and sex) and assessment of outcome, were assessed and scored as ‘0’ (not appropriate), ‘1’ (appropriate) or ‘N/A’ (not applicable). A rating from 0 to 5 was assigned to each study and subsequently divided by the total number of variables assessed. A final rating from 0 to 1 was assigned with higher scores indicating a lower risk of bias (Table S2 in the **Online Supplementary Document**).

### Statistical analysis

Random effects meta-analysis was used to pool data on the percentages of physical inactivity and physical activity, and people engaging in sedentary behaviour using generic inverse variance methods [9]. Results were presented as percentage values and 95% confidence intervals, and forest plots were generated for each analysis. Estimates from studies that reported a direct comparison between rural vs urban and less remote vs more remote populations were pooled into a subgroup analysis. We focused our interpretation of results on the best estimate (pooling effect) and relevant confidence intervals rather than on hypothesis or statistical inference. Review Manager (RevMan) version 5.3 (The Nordic Cochrane Centre, The Cochrane Collaboration, 2014. Copenhagen, Denmark) was used for all analysis.

## RESULTS

**Figure 1:**
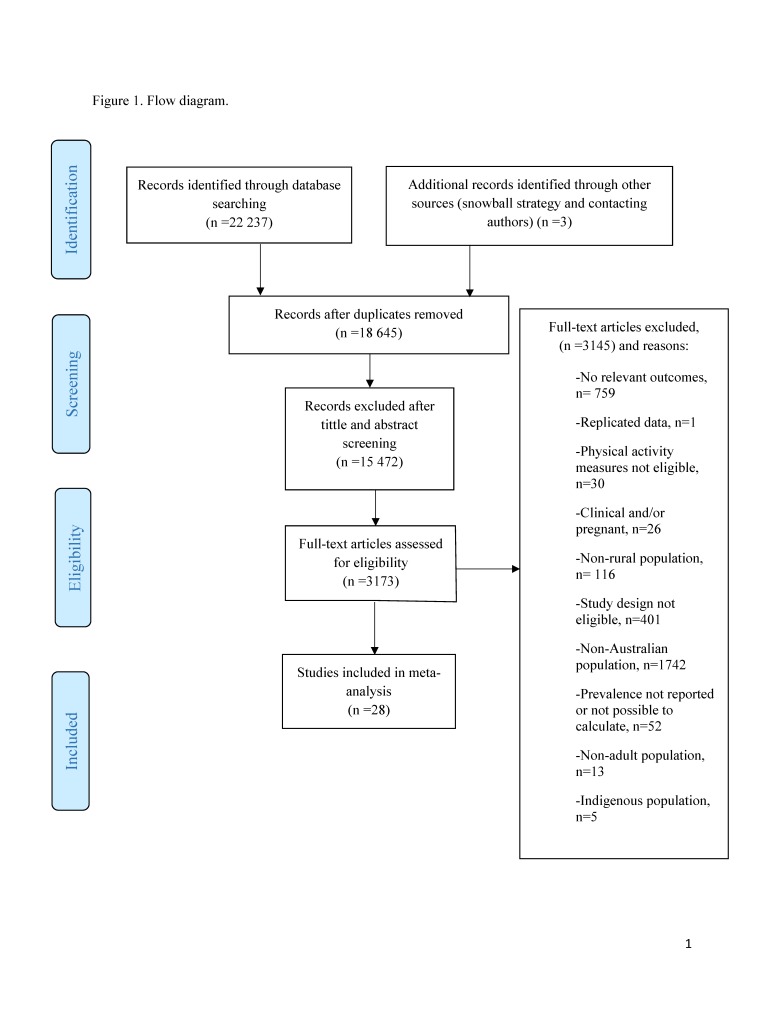
Flow diagram.

A total of 22 237 studies were identified. We excluded 3592 duplicates, 9187 studies in the title screening phase, and 6285 studies during the abstracts screening phase. Thus, 15 472 titles and abstracts were excluded. After the full-text screening, 28 studies [8,10-36] fulfilled the inclusion criteria (**Figure 1**). Seven cohorts and 21 cross-sectional studies published between 2004 and 2017 were included. Studies included a total of 51 5532 Australian adults; 21 995 from a rural or remote area, and 296 037 from urban regions. The age range across the included studies was 18 to 93 years old, with seven studies including only women in their samples (**Table 1**). A total of 15 studies (54%) assessed physical activity subjectively with the Active Australia Survey or the long form of the International Physical Activity Questionnaire (IPAQ-L) [8,11,13-17,19,21,23,25-29], meanwhile only three studies (11%) reported measures using accelerometers [28,32,35]. Seven studies (25%) that assessed either physical activity or sedentary behaviour using a non-specified questionnaire or a questionnaire other than the Active Australia Survey or IPAQ were published up to 2010. Regarding the definitions of rural settings, 12 studies (43%) did not describe which classification system was used to determine rurality or remoteness, and 14 studies (50%) provided physical activity data on both rural and urban populations. The study quality score using the modified NOS ranged from 0.25 to 1.00 (higher scores indicate a lower risk of bias), with an average of 0.7 (Table S2 in the **Online Supplementary Document**), and representing an overall low risk of bias for the outcomes assessed in the included studies.

**Table 1:** Study and sample characteristics of the studies included

Study	Study design	Rural sample size	Urban sample size	Age (mean)	Gender (% female)	Physical assessment method	Rural definition	Levels of remoteness or rurality
Aird and Buys 2015 [10]	Cross-sectional	24	24	73	50%	Self-reported questionnaire	N/R	Inner city, city suburban, regional city and rural town
Badland et al. 2008 [11]	Cross-sectional	765	N/A	N/R	47%	Active Australia	The regional, remote, and metropolitan areas classification (RRMA, 2005)	Large rural, small rural and remote
Ball et al. 2004* [12]	Cross-sectional	6628	4014	N/R	100%	Self-reported questionnaire	Rural, Remote and Metropolitan Areas Classification (RRMA, 1991)	Urban, rural and remote
Ball et al. 2013* [13]	Cross-sectional	2184	1882	N/R	100%	International Physical Activity Questionnaire- Long version (IPAQ-L)	The Socio-Economic Index for Areas (SEIFA Index of Disadvantage, 2001)	Urban and rural
Berry et al. 2017 [14]	Cross-sectional	664	1738	49	52%	Active Australia	Australian Bureau of Statistics remoteness classification of urban, rural and remote, 2000	Urban and rural
Brown et al. 2013 [15]	Cross-sectional	1219	N/A	46	58%	Active Australia	Australian Standard Geographic Classification (ASGC, 2011)	Rural
Carroll et al. 2014 [16]	Cross-sectional	290	N/A	48	60%	Active Australia	N/R	Rural
Cleland et al. 2010 [17]	Cross-sectional	2179	1844	34	100%	International Physical Activity Questionnaire- Long version (IPAQ-L)	The Socio-economic Index for Areas (SEIFA Index of Disadvantage, 2001)	Urban and rural
Cole et al. 2006 [18]	Cross-sectional	815	2576	N/R	51%	Self-reported questionnaire	Australian Bureau of Statistics, 1991.	Urban and rural
Dalbo et al. 2015 [19]	Cross-sectional	1289	N/A	N/R	51%	Active Australia	N/R	Rural
Davis-Lameloise et al. 2013 [20]	Cross-sectional	1001	N/A	51	52%	Self-reported questionnaire	N/R	Rural
Ding et al. 2014 [21]	Cohort	105 889	85 803	N/R	N/R	Active Australia	N/R	Urban and rural
Dobson et al. 2010 [22]	Cohort	7650	4750	73	100%	Self-reported questionnaire	Australian Standard Geographic Classification (ASGC)	Major city, inner regional, outer regional and remote
Duncan et al. 2009 [23]	Cross-sectional	532	676	N/R	52%	Active Australia	Rural, Remote and Metropolitan Areas Classification (RRMA, 2005)	Urban and rural
Eime et al. 2014 [24]	Cross-sectional	710	N/A	39	100%	Self-reported questionnaire	N/R	Rural
Eley et al. 2014 [25]	Cross-sectional	2000	N/A	N/R	N/R	Active Australia	Australian Bureau of Statistics 2006.	Rural
George et al. 2012 [26]	Cross-sectional	3387	14 286	N/R	N/R	Active Australia	The Accessibility/Remoteness Index of Australia (ARIA+) 2004	Urban and rural
Harrison et al. 2017 [27]	Cohort	576	N/A	40	100%	International Physical Activity Questionnaire- Long version (IPAQ-L)	Rural Victorian towns 100–400km from the state capital, Melbourne, and with a population of 2000–10 000 residents	Rural
Patterson et al. 2015 [28]	Cross-sectional	367	982	N/R	N/R	International Physical Activity Questionnaire- Long version (IPAQ-L) and pedometer	The Accessibility/Remoteness Index of Australia (ARIA+) 2006	Urban and rural
Pontt et al. 2015 [35]	Cross-sectional	58	N/A	49	0%	Accelerometer activPAL monitor	Accessibility/Remoteness Index of Australia (ARIA) and (Australian Bureau of Statistics, 2006b).	Rural
Powers et al. 2017a* [36]	Cohort	6840	7358	N/R	100%	Self-reported questionnaire	N/R	Urban and rural
Powers et al. 2017b* [36]	Cohort	4181	12 677	N/R	100%	Self-reported questionnaire	N/R	Urban and rural
Sealey et al. 2010 [29]	Case study, cohort	196	132	39	84%	Active Australia	(Australian Institute of Health and Welfare, 2004)	Urban, rural and remote
Simmons et al. 2005 [30]	Cross-sectional	1454	N/A	53	56%	Self-reported questionnaire	Australian Bureau of Statistics, 2001	Regional centre, large rural and small rural
Simmons et al. 2007 [31]	Cross-sectional	495	N/A	56	53%	Self-reported questionnaire	N/R	Rural
Sushames et al. 2015 [32]	Cohort	36	N/A	33	N/R	Self-reported questionnaire and accelerometer	N/R	Rural
Van der Ploeg et al. 2012 [8]	Cohort	65 018	157 295	N/R	N/R	Active Australia	N/R	Urban and rural
Vaughan et al. 2008 [33]	Cross-sectional	1509	N/A	55	53%	Self-reported questionnaire	N/R	Rural
Vaughan et al. 2009 [34]	Cross-sectional	1539	N/A	55	47%	Self-reported questionnaire	N/R	Rural

N/A – not assessed, N/R – not reported

*Data provided independently by the study author.

### Prevalence of physical inactivity in rural and urban populations

The prevalence of physical inactivity – percentage of rural Australian adults not meeting physical activity recommendations according to the WHO guidelines [1] was reported in 19 studies [10-19,21,22,24,25,27-29,33,36] (Powers et al [36] included two cohorts in the same article). Among these, 11 studies [10,12-14,17,18,21,22,28,29,36] also provided data on the physical activity of Australian adults living in urban areas, allowing for a direct comparison across rural and urban populations. The most common threshold used to classify people not meeting physical activity recommendations was the accumulation of 0 to 149 minutes of physical activity per week.

**Figure 2:**
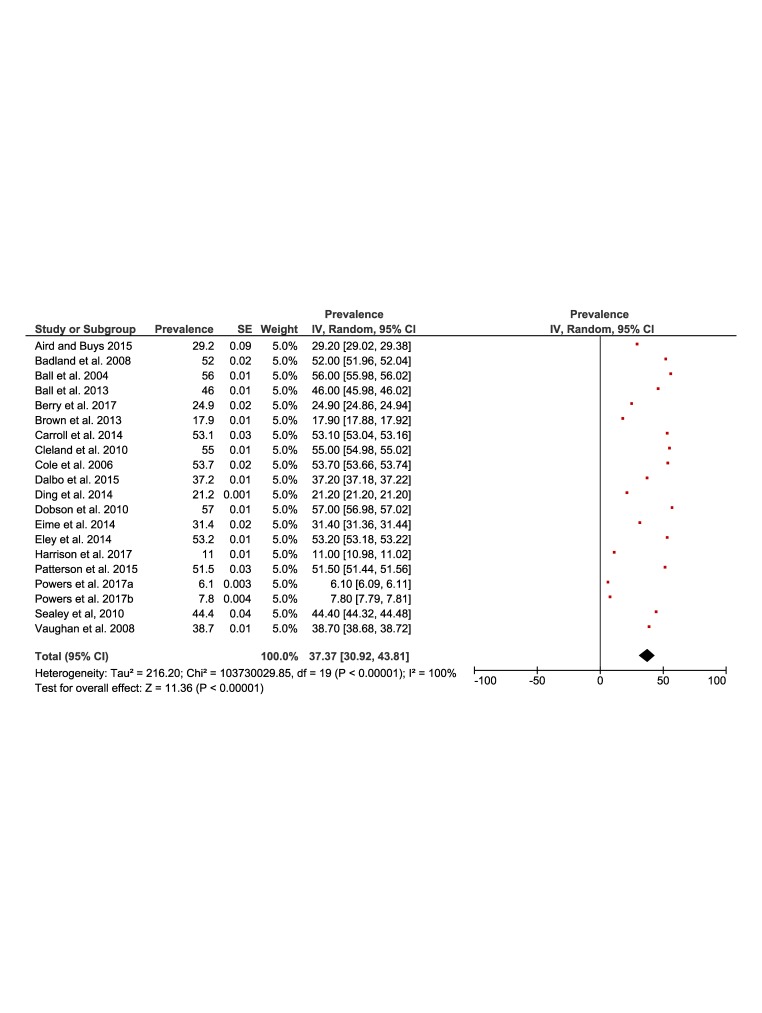
Forest plot of the prevalence of physical inactivity of the rural population.

**Figure 3:**
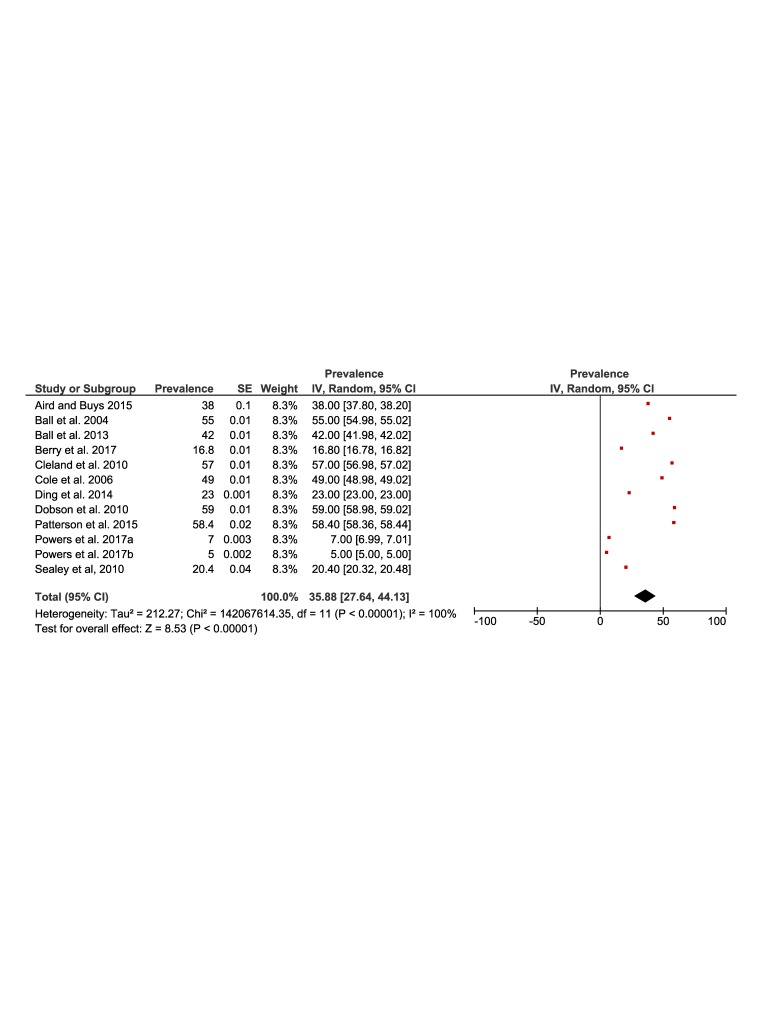
Forest plot of the prevalence of physical inactivity of the urban population.

**Figure 2** and **Figure 3** present the results of the meta-analysis of the prevalence of physical inactivity in rural (37%, 95% CI = 30 to 43) and urban (35%, 95% confidence interval (CI) = 27 to 44) settings, respectively. The pooling of rural populations (**Figure 2**) included a total of 19 studies reporting data on 145 975 people. The minimum prevalence value was 6.1% [36] and the maximum was 57% [22]. The pooling of urban populations (**Figure 3**) included 11 studies with a total population of 123 780 people. The minimum prevalence value was 5% [36] and the maximum was 59% [22].

**Figure 4:**
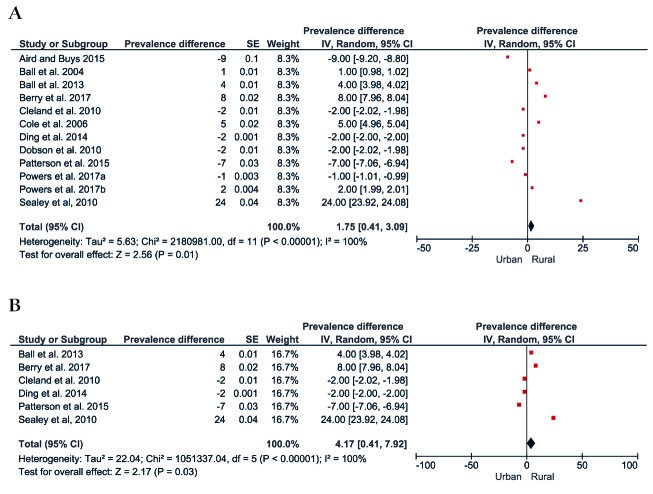
Prevalence of physical inactivity in Australia. **Panel A.** Forest plot of the prevalence difference of physical inactivity of the rural vs urban population. **Panel B.** Forest plot showing a sensitive analysis of the prevalence difference of physical inactivity of the rural vs urban population including only studies that used Active Australia Survey or IPAQ.

**Figure 4** shows the forest plot of the prevalence difference of physical inactivity between rural and urban populations. A total of 11 studies comprising 269 755 rural and urban dwelling Australians were included, with pooling showing rural populations with a slightly higher prevalence of physical inactivity (1%, 95% CI = 0.4 to 3, see **Figure 4****, **Panel A). However, a post-hoc sensitivity analysis including only studies that assessed physical activity using the Active Australia Survey or the IPAQ, showed a four percentage point (95% CI = 0.4 to 7) greater prevalence of physical inactivity in rural populations compared to urban populations (**Figure 4**, Panel B).

### Prevalence of physical activity in rural and urban populations

The prevalence of physical activity – percentage of rural populations meeting physical activity recommendations according to the WHO guidelines [1] was reported in 21 studies [10-13,15-20,22-24,26-28,30-34]. Among these, nine studies [10,12,13,17,18,22,23,26,28] also provided data on urban people allowing for a direct comparison between the rural and urban populations.

The most common threshold used to classify people meeting physical activity recommendations was the accumulation of ≥150 minutes of physical activity per week, however, in nine studies [10,12,20,22,26-28,33,34] the classification system was either different from the accumulation of ≥150 minutes of physical activity per week (eg, moderate to vigorous physical activity ≥4 times per week), or not defined (eg, self-reported coded as active and very active). Five studies [10,13,22,31,32] did not report the domain and/or intensity of physical activity, meanwhile, four studies [11,18,26,30] reported measures on physical activity regarding walking, recreation, and transport conjointly, and six studies [12,22,27,30,33,34] focused only in moderate and/or vigorous physical activity. These studies were included in the meta-analysis and categorised as meeting physical activity recommendations for meta-analysis purposes.

**Figure 5:**
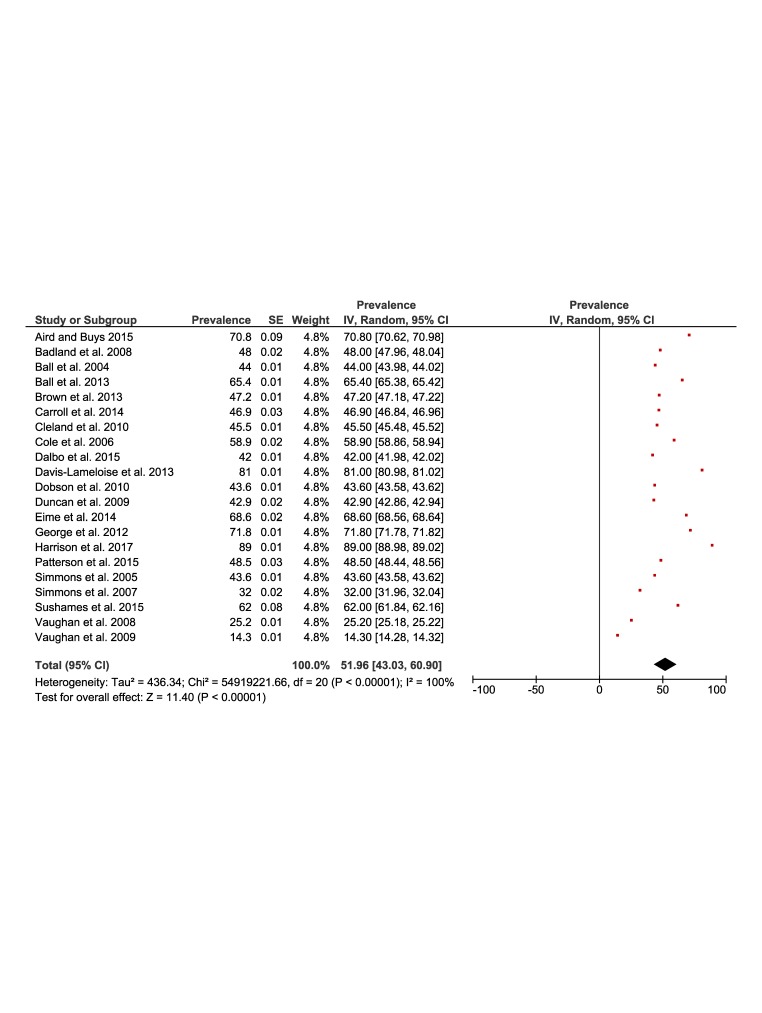
Forest plot of the prevalence of physical activity of the rural population.

**Figure 6:**
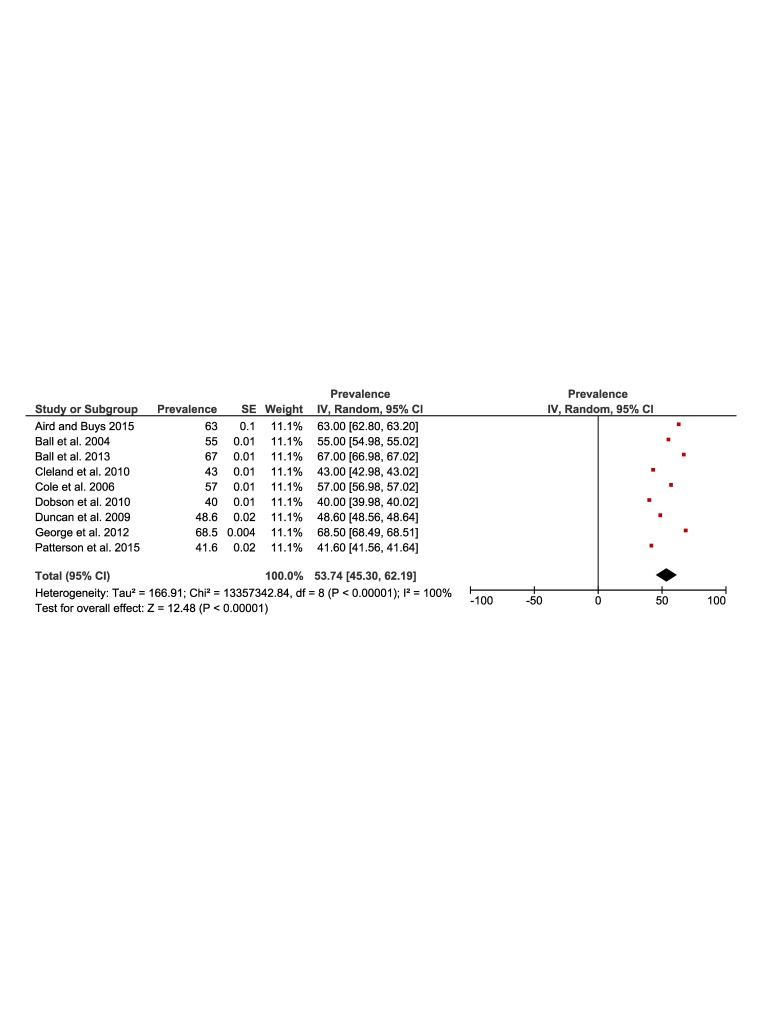
Forest plot of the prevalence of physical activity of the urban population.

**Figure 5** and **Figure 6** present the results of the meta-analysis of the prevalence of physical activity in rural (51%, 95% CI = 43 to 60) and urban (53%, 95% CI = 45 to 62) populations. The pooling of the rural populations (**Figure 5**) included a total of 21 studies reporting data on 34 649 people. The minimum prevalence value was 14% [34] and the maximum was 89% [27]. The pooling of urban populations **(****Figure 6****)** included nine studies reporting data on 31 034 people. The minimum prevalence value was 40% [22] and the maximum was 68% [26].

**Figure 7:**
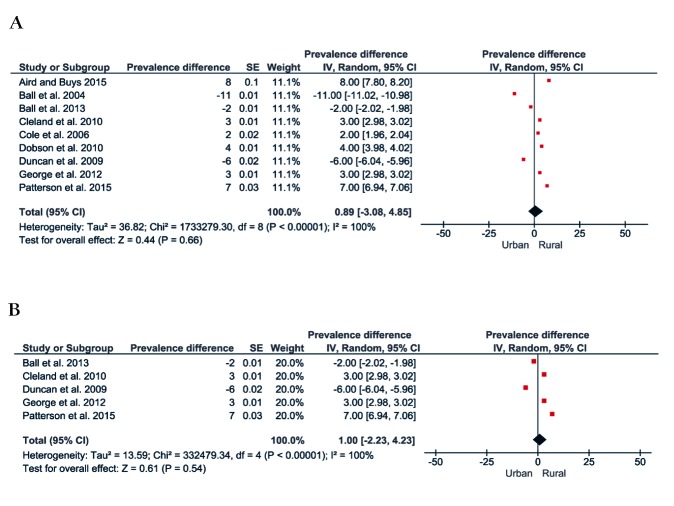
Prevalence of physical activity in Australia. **Panel A.** Forest plot of the prevalence difference of physical activity of the rural vs urban population. **Panel B.** Forest plot showing a sensitive analysis of the prevalence difference of physical activity of the rural vs urban population including only studies that used active Australia or IPAQ.

**Figure 7**, Panel A shows the forest plot of the prevalence difference of physical activity of nine studies with a total population of 65 683 rural and urban Australian adults. There was a one percentage point (95% CI = -3 to 5) greater prevalence of physical activity in favour of rural populations. The results were similar in those studies that assessed physical activity using the Active Australia Survey or IPAQ (**Figure 7**, Panel B).

### Prevalence of sedentary behaviour in rural and urban populations

The meta-analysis of sedentary behaviour in rural and urban populations included five studies. High levels of sedentary behaviour was defined in one study as spending ≥8 hours sitting per day [21], meanwhile, two studies used time spent in reading, watching TV, or in other passive pursuits [33,34]. Only one study defined high sedentary behaviour as more than 720 minutes per week watching television [30]. One study categorized sedentary behaviour in four categories (time spent in sitting: 0 to <4; 4 to <8; 8 to <11 and ≥11 hours per day) [8].

**Figure 8:**
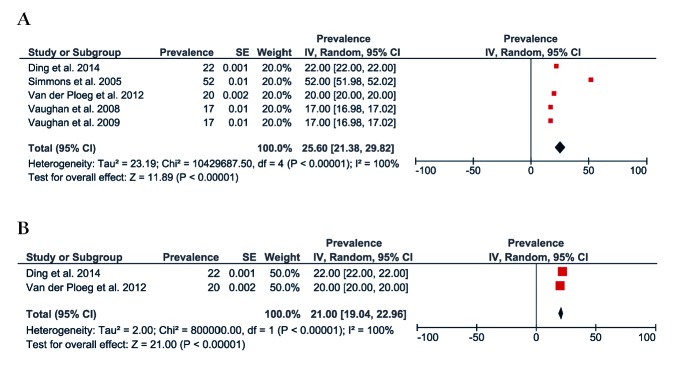
Prevalence of sedentary behaviour in rural Australia. **Panel A.** Forest plot of the prevalence of rural population reporting sedentary behaviour. **Panel B.** Forest plot showing a sensitive analysis of the prevalence of sedentary behaviour in rural population only with studies that defined sedentary behaviour as spending 8 hours sitting per day.

**Figure 9:**
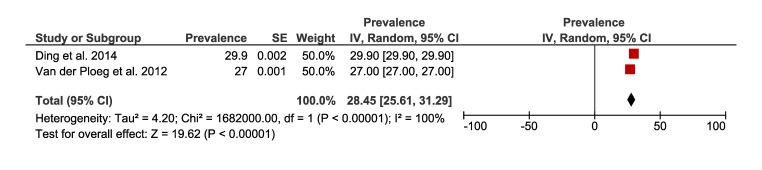
Forest plot of the prevalence of urban population reporting sedentary behaviour.

**Figure 8** and **Figure 9** present the results of the meta-analysis including five studies that reported the prevalence of sedentary behaviour of rural (25%, 95% CI = 21 to 29) and urban (28%, 95% CI = 25 to 31) populations. The pooling of rural populations (**Figure 8**, Panel A) included a total of 17 5432 people. A post-hoc sensitivity analysis only including studies that assessed sedentary behaviour in rural populations defined as spending ≥8 hours sitting per day, showed a pooled prevalence of 21% (95% CI = 19 to 22), which is slightly smaller compared to the original analysis (**Figure 8**, Panel B). The pooling of urban populations (**Figure 9**) included two studies with a total population of 243 098 people.

**Figure 10:**
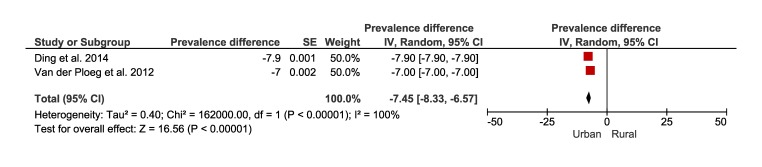
Forest plot of the prevalence difference of rural vs urban population reporting sedentary behaviour.

**Figure 10** shows the forest plot for two studies with a total of 418 530 people comparing sedentary behaviour between rural and urban Australian adults. The prevalence of sedentary behaviour was slightly greater in those living in urban areas compared to rural areas (-7%, 95% CI = -8 to -6).

**Figure 11:**
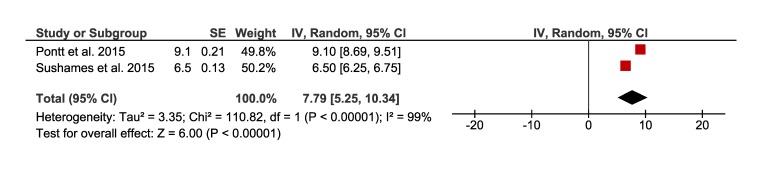
Forest plot of pooling of overall average of hours per day in sedentary time measured with accelerometer in rural population.

**Figure 11** present the meta-analysis and the pooling of sedentary time assessed with accelerometry in a total sample of 94 rural Australian adults. Results show that rural populations spend an overall average of 7.8 hours per day (95% CI = 5 to 10) engaged in sedentary time.

### Prevalence of physical inactivity according to levels of geographical remoteness

**Figure 12:**
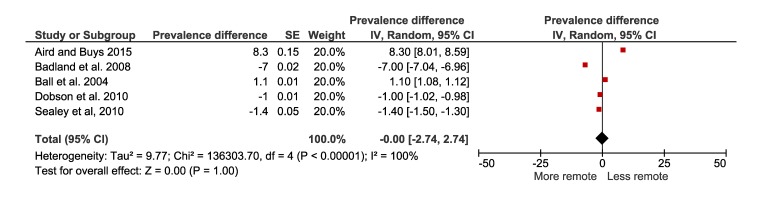
Forest plot of prevalence difference of physical inactivity of less remote vs more remote population.

The meta-analysis on the prevalence of physical inactivity of the less remote and more remote populations included five studies with a total of 15 263 (**Figure 12**). No difference was observed in the prevalence of physical inactivity between people living in less remote or more remote areas (0%, 95% CI = -2 to 2).

### Prevalence of physical activity according to levels of geographical remoteness

**Figure 13:**
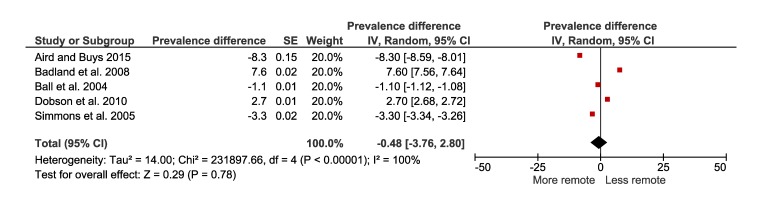
Forest plot of prevalence difference of physical activity of remote vs very remote population.

The meta-analysis on the prevalence of less remote and more remote populations meeting physical activity recommendations included five studies with a total of 16 521 people (**Figure 13**) and showed that the percentage of people being active is slightly greater in people living in more remote areas (0.5% 95% CI = -4 to 3).

## DISCUSSION

This systematic review and meta-analysis summarised the prevalence of physical activity, inactivity, and sedentary behaviour of people who live in rural areas, using Australian adult populations. Although we found that rural dwellers spend slightly less time in a sedentary behaviour (eg, engaging less frequently in high volumes of time with a low energy expenditure in a sitting or reclining posture during wake times) when compared with urban dwellers, we found a higher prevalence of physical inactivity (eg, lack of physical activity and/or not meeting moderate to vigorous physical activity recommendations) in rural Australian adults compared to their urban counterparts. Meanwhile, we found that people living in more remote areas had similar levels of physical inactivity compared with people in less remote areas.

### Prevalence of physical inactivity

Our results are in line with a government report published by the Australian Institute of Health and Welfare [5], which showed that 60% of adults aged 18 and over were not sufficiently active in 2014-2015, given that they are not meeting the physical activity levels recommended by WHO. According to the Australian Institute of Health and Welfare report [5], 60% of people living in rural and 50% of people living in urban areas do not meet physical activity recommendations and were deemed insufficiently active.

The factors associated with physical inactivity may vary between populations (ie, rural or urban) and could be related to air pollutants, built environment (eg, distance to parks, road safety), and sociodemographic characteristics (eg, access to service, socioeconomic status, food quality, educational levels, and crime) [5,37]. We hypothesize that factors associated with rural lifestyles such as higher rates of tobacco smoking and alcohol consumption in comparison with urban lifestyles [5], also play an important role and could be a possible explanation for the prevalence of physical inactivity in this population.

Although our study did not focus on the differences between domains of physical activity (eg, leisure time, commuting, domestic or transport), previous studies highlighted that people living in rural areas acquire more physical activity in domains such as occupational and domestic physical activity, meanwhile, urban populations tend to accumulate greater physical activity through leisure and commuting to and from work [28]. Rurality may reflect a less active approach to commuting to and from work possibly because of large distances, less infrastructure for commuting, and concerns around road safety. In addition, many people living in rural areas work where they reside (eg, farming), so commuting is not required [33]. It is also suggested that infrastructure planning and transportation policies are possible strategies to encourage people to have a more active lifestyle, however, implementing community-based physical activity programs in rural communities is challenging due to limited access to services and facilities [38].

### Prevalence of sedentary behaviour in Australian adults

Although there is not a consensus regarding the daily cut-off values of sedentary time associated with increased mortality and disease incidence, a study published in 2018 with a meta-regression including more than one million participants suggests that nine hours per day or more of sedentary behaviour is associated with increased risk of mortality (hazard ratio = 1.22) in adults [39]. Our meta-analysis including two studies [32,35] that assessed sedentary time with accelerometers, found that the average sedentary time per day was 7.8 hours among the rural population. Similar results were found in a study that included data from 10 countries with populations from city-regions, which concluded that the average sedentary time per day, assessed through accelerometry, was 8.5 hours [40].

There is limited information on the level of sedentary behaviour of populations living in rural settings. Our results showed that Australians living in urban areas have a higher prevalence of sedentary behaviour when compared with those living in rural areas, with a prevalence difference of 7 percentage points (95% CI = 8 to 7). This is in line with other reports [41] that showed that urbanisation is associated with increased sedentary behaviour due to sedentary jobs, electronic entertainment, the use of labour-saving devices at home, as well as passive modes of commuting [42]. Meanwhile, a study published in 2010 [43] found that men and women from rural areas were more likely to watch 2 hours of television per day or more (odds ratio 1.34 in men and 1.51 in women) compared to urban Australians, which also was associated with social disadvantage and older age. We hypothesized that the differences between rural and urban populations regarding sedentary behaviour could be related to the common occupations in these settings - for example, agricultural and farming occupations in rural areas implying large amounts of time spent in passive pursuits during the occupational time. However, a 2016 report on regional Australia [44] showed that agricultural, forestry, and fishing made up only 6.5% of employed people. Therefore, further research is needed in order to elucidate the factors associated with sedentary behaviour in rural populations.

### Prevalence of physical activity in Australian adults

The results of our meta-analysis showed that 52% of the rural populations and 54% of the urban populations met physical activity guidelines, which is in line with an Australian government report [45] showing that 48% of adults aged 18-64 years old meet physical activity guidelines. Some aspects that could be related to greater physical activity engagement in rural areas include the occupational and domestic physical activity (eg, larger properties yard and physically demanding jobs) [28]. High levels of physical activity found in some studies could be related to: (i) more occupational and domestic physical activity of rural population than those living in urban areas [28]; (ii) rural communities with access to active environments such as parks and walking paths, but it cannot be generalised since rural areas are commonly associated with fewer neighbourhood environmental supports for physical activity and low-quality parks [46]. Only one study described occupational physical activity levels in rural Australia; Vaughan et al [33] reported that from a sample of 490 men, 220 (45%) were involved in agriculture, forestry or fishing occupations and 66% reported their occupation physical activity level as high.

### Strategies for the promotion of physical activity

Specific physical activity-promoting interventions to support urban dwellers commonly target specific barriers such as the lack of information and education regarding physical activity; behavioural and social interventions for lifestyle change; and environmental and policy approaches that enhance supportive environments and promote activities in the community. These types of strategies are recommended and have proved effective to successfully increase physical activity behaviours [47,48]. Although there appear to be setting-specific barriers to physical activity in rural areas, such as lack of sports infrastructure and public parks [46], there is a paucity of interventions addressing these barriers^[^49^]^. Additionally, it is unclear if the interventions aimed at increasing physical activity in urban settings are scalable to rural locations [47].

### Strength and limitations

This systematic review is the first, to our knowledge, to summarise and provide robust, quantitative data using a meta-analytical approach on physical activity, inactivity, and sedentary behaviour in rural populations, using Australia as the context. We prospectively registered the protocol of the systematic review, used a sensitive search strategy, and identified 28 studies that were included in a meta-analysis. We assessed the risk of bias of the studies included and performed a sensitivity analysis with only studies that used Active Australia Survey or IPAQ to assess physical activity and/or inactivity.

Some limitations of this study need to be taken into account when interpreting the results. The analysis of subjective self-report measures, which are susceptible to recall bias of physical activity and sedentary behaviour, could affect results since people tend to provide socially desirable answers (ie, increasing activity time and reducing sedentary behaviour time). However, this is the nature of large observational studies of physical activity and a limitation difficult to be overcome. Although most of the studies in our systematic review included populations from different states and regions of Australia, we cannot discard the possibility of case overlapping (ie, the same participant could be included in two studies). The definitions of physical activity, inactivity, and sedentary behaviour had some variations, as well as the type and/or intensity of physical activity was not homogeneous across the included studies. Only two of the studies included used an objective tool to assess sedentary behaviour, however, this is not surprising since most population-based studies tend to use self-reported measures to assess physical activity. Although the use of accelerometer provides more accurate estimates of sedentary time, waist (which is the case of one of the studies) and wrist-worn accelerometers could not detect the difference between standing and sitting. Most of the studies included in the meta-analysis assessed physical activity occurring in leisure-time and transportation domains, and therefore, it may not reflect the engagement of rural populations in occupational physical activity.

The weight of the conclusions about physical activity participation based on the population’s level of rurality should also consider evidence from national representative surveys, which commonly employ rigorous sampling methods and consistent physical activity measurement methodology, although most of these surveys employ self-reported tools.

## CONCLUSIONS

The percentage of adults not meeting current physical activity recommendations is as high in rural as it is in urban areas. Our results challenge the notion that people living in rural areas are physically active. Studies assessing physical activity and sedentary behaviour objectively (eg, using thigh-worn accelerometers) are needed for better comparisons on physical activity or sedentary behaviour association of rurality. Public health campaigns promoting physical activity in rural settings are as necessary as in urban settings.

## Additional material

Online Supplementary Document
