# Supplementary material for: Are people in the bush really physically active? A systematic review and meta-analysis of physical activity and sedentary behaviour in rural Australians populations
Source: J Glob Health. 2020 Apr 15;10(1):010410. doi: 10.7189/jogh.10.010410 (PMC7182355; doi:10.7189/jogh.10.010410)
Supplement: Online Supplementary Document [file jogh-10-010410-s001.pdf]

Are people in the bush really physically active? A systematic review and meta-analysis of physical activity and sedentary behaviour in rural Australians populations

Ovid MEDLINE(R)

	Search terms	N
#1	exp exercise/ OR exp exercise therapy/ OR "physical education and training"/ OR (physic* adj (activ* OR fit* OR exercis*)).mp. OR (walk* OR jog* OR swim* OR weight lift* OR danc* OR aerobics).mp. OR ((physic* OR strength* OR resist* OR circuit OR weight OR aerob* OR cross OR endurance OR structur*) adj3 train*).mp. OR sedentary Lifestyle/ OR physical activity in leisure time.mp. OR (physical* adj inactiv*).mp. OR ((sedentary OR inactive) and (life* OR behav* OR life style)).mp.	419722
#2	exp rural health/ OR exp rural population/ OR ((rural* OR remote* OR nonmetropolitan OR non metropolitan OR village OR provinc* OR non-urban OR nonurban OR outback OR country side OR countryside OR agrarian OR agricultural OR farm*) adj (population OR demographic OR dweller* OR citizen* OR inhabitant* OR communit* OR people OR resident* OR societ*)).mp.	84362
#3	#1 AND #2	3,072

Embase (via OvidSP)

	Search terms	N
#1	exp exercise/ OR physical education/ OR exp “physical activity, capacity and performance”/ OR exp kinesiotherapy/ OR (physic* adj (activ* OR fit* OR exercis*)).mp. OR (walk* OR jog* OR swim* OR weight lift* OR danc* OR aerobics).mp. OR ((physic* OR strength* OR resist* OR circuit OR weight OR aerob* OR cross OR endurance OR structur*) adj3 train*).mp. OR physical activity in leisure time.mp. OR (physical* adj inactiv*).mp. OR sedentary lifestyle/ OR sitting/ OR ((sedentary OR inactive) and (life* OR behav* OR life style)).mp.	953327
#2	exp rural area/ OR rural population/ OR rural* OR remote* OR nonmetropolitan OR non metropolitan OR village* OR provinc* OR non-urban OR nonurban OR outback OR country side OR countryside OR agrarian OR agricultural OR farm*) adj (population OR demographic OR dweller* OR citizen* OR inhabitant* OR communit* OR people OR resident* OR societ*)).mp	91419
#3	#1 AND #2	6,193

CINAHL

	Search terms	N
#1	(MH "Exercise+") OR (MH "Therapeutic Exercise+") OR (MH "Physical Education and Training+") OR TX (physic* N1 (activ* OR fit* OR exercis*) OR TX (walk* OR jog* OR swim* OR weight lift* OR danc* OR aerobics) OR TX ((physic* OR strength* OR resist* OR circuit OR weight OR aerob* OR cross OR endurance OR structur*) N3 train*) OR TX physical activity in leisure time OR TX (physical* N1 inactiv*) OR (MH "Life Style, Sedentary") OR (MH "Sitting") OR TX ((sedentary OR inactive) and (life* OR behav* OR life style))	168,876
#2	(MH "Rural Health") OR (MH "Rural Population") OR TX (rural* OR remote* OR nonmetropolitan OR non metropolitan OR village* OR provinc* OR non-urban OR nonurban OR outback OR country side OR countryside OR agrarian OR agricultural OR farm*) N1 (population OR demographic OR dweller* OR citizen* OR inhabitant* OR communit* OR people OR resident* OR societ*))	12,935
#3	#1 AND #2	1,234

AMED

	Search terms	N
#1	exp exercise/ OR exp exercise therapy/ OR physical education/ OR (physic* adj (activ* OR fit* OR exercis*)).mp. OR (walk* OR jog* OR swim* OR weight lift* OR danc* OR aerobics).mp. OR ((physic* OR strength* OR resist* OR circuit OR weight OR aerob* OR cross OR endurance OR structur*) adj3 train*).mp. OR sedentary Lifestyle/ OR physical activity in leisure time.mp. OR (physical* adj inactiv*).mp. OR ((sedentary OR inactive) and (life* OR behav* OR life style)).mp.	29718
#2	exp rural health/ OR exp rural population/ OR ((rural* OR remote* OR nonmetropolitan OR non metropolitan OR village OR provinc* OR non-urban OR nonurban OR outback OR country side OR countryside OR agrarian OR agricultural OR farm*) adj (population OR demographic OR dweller* OR citizen* OR inhabitant* OR communit* OR people OR resident* OR societ*)).mp.	601
#3	#1 AND #2	24

Sportdiscus

	Search terms	N
#1	TX exercise OR TX therapeutic exercise OR TX physical education and training OR TX (physic* N1 (activ* OR fit*) OR TX (walk* OR jog* OR swim* OR weight lift* OR danc* OR aerobics) OR TX ((physic* OR strength* OR resist* OR circuit OR weight OR aerob* OR cross OR endurance OR structur*) N3 train*) OR TX physical activity in leisure time OR TX (physical* N1 inactiv*) OR TX sedentary lifestyle OR TX ((sedentary OR inactive) and (life* OR behav* OR life style))	644,415

#2	TX rural health OR TX rural population OR TX rural area OR TX (rural* OR remote* OR nonmetropolitan OR non metropolitan OR village* OR provinc* OR non-urban OR nonurban OR outback OR country side OR countryside OR agrarian OR agricultural OR farm*) N1 (population OR demographic OR dweller* OR citizen* OR inhabitant* OR communit* OR people OR resident* OR societ*))	14,552
#3	#1 AND #2	8,529

Rural and Remote Health Database via Informit Online (1996 - 2006)

	Search terms	N
#1	(("physical inactivity") OR ("sedentary lifestyle") OR sedentary OR inactive) OR (strength OR aerobic OR endurance OR circuit OR resistance) OR (("physical fitness") OR training OR swimming OR dance OR walk) OR (exercise OR ("therapeutic exercise") OR ("physical education") OR therapy OR ("physical activity"))	4,580
#2	(("country side") OR countryside OR agrarian OR agricultural OR farm) OR (province OR nonurban OR ("non urban") OR "non-urban" OR outback) OR (rural OR remote OR nonmetropolitan OR ("non metropolitan") OR village) OR (("rural health") OR ("rural population") OR ("rural area"))	25,808
#3	#1 AND #2	3,182

Are people in the bush really physically active? A systematic review and meta-analysis of physical activity and sedentary behaviour in rural Australians populations

	**Selection**		**Comparability**		**Outcome**			
Author	**Representativeness of exposed cohort**	**Selection of non-exposed cohort**	**Adjust for age**	**Adjust for other risk factors**	**Assessment of outcome**	**Points**	**Outcomes assessed**	**Quality score**
Aird and Buys 2015 [10]	**0**	**1**	**0**	**0**	**1**	**2**	**5**	**0.4**
Badland et al. 2008 [11]	**1**	N/A	**1**	**1**	**1**	**4**	**4**	**1**
Ball et al. 2004 [12]	0	1	1	0	1	3	5	0.6
Ball et al. 2013 [13]	0	1	1	0	1	3	5	0.6
Berry et al. 2017 [14]	1	1	1	1	1	4	4	1
Brown et al. 2013 [15]	0	N/A	1	1	1	3	4	0.75
Carroll et al. 2014 [16]	1	N/A	1	1	1	4	4	1
Cleland et al. 2010 [17]	0	1	1	1	1	4	5	0.8
Cole et al. 2006 [18]	1	1	1	1	1	5	5	1
Dalbo et al. 2015 [19]	1	N/A	1	1	1	4	4	1
Davis-Lameloise et al. 2013 [20]	1	N/A	1	1	1	4	4	1
Ding et al. 2014 [21]	1	1	1	1	1	5	5	1
Dobson et al. 2010 [22]	0	0	1	0	1	2	5	0.4
Duncan et al. 2009 [23]	1	1	1	1	1	5	5	1
Eime et al. 2014 [24]	0	N/A	1	0	1	2	4	0.5

	Selection		Comparability		Outcome			
Author	**Representativeness of exposed cohort**	**Selection of non-exposed cohort**	**Adjust for age**	**Adjust for other risk factors**	**Assessment of outcome**	**Points**	**Outcomes assessed**	**Quality score**
Eley et al. 2014 [25]	0	N/A	0	0	1	1	4	0.25
George et al. 2012 [26]	0	1	1	1	1	4	5	0.8
Harrison et al. 2017 [27]	0	N/A	0	1	1	2	4	0.5
Patterson et al. 2015 [28]	0	1	1	1	1	4	5	0.8
Pontt et al. 2015 [35]	0	N/A	1	1	1	3	4	0.75
Powers et al. 2017 [36]	0	1	1	1	1	4	5	0.8
Sealey et al. 2010 [29]	0	1	0	1	1	3	5	0.6
Simmons et al. 2005 [30]	1	N/A	1	1	1	4	4	1
Simmons et al. 2007 [31]	1	N/A	1	0	1	4	4	1
Sushames et al. 2015 [32]	0	N/A	0	0	1	1	4	0.25
Van der Ploeg et al. 2012 [8]	0	1	1	1	1	4	5	0.8
Vaughan et al. 2008 [33]	1	N/A	1	1	1	4	4	1
Vaughan et al. 2009 [34]	1	N/A	1	1	1	4	4	1
								0.7
